# Global Myocardial Work Efficiency and Left Ventricular Ejection Fraction After Left Bundle Branch Area Pacing Versus Right Ventricular Pacing: Extended Follow-Up of a Prospective Cohort

**DOI:** 10.3390/jcm15218224

**Published:** 2026-10-22

**Authors:** Georgios Leventopoulos, Maria Bozika, Panagiotis Chronopoulos, Eleni Papastavrou, Christos Kyriazis, Kassiani-Maria Nastouli, Georgios Boliaris, Panagiotis Patrinos, Angelos Perperis, Rafail Koros, Ioanna Koniari, Georgia Xygka, Efthimia Kapsali, Anastasios Apostolos, Nikolaos Vythoulkas-Biotis, Alexandros Dedes, Anastasia Efraimoglou, Athanasios Moulias, Periklis Davlouros

**Affiliations:** 1 Department of Medicine, Division of Cardiology, University Hospital of Patras, 265 04 Patras, Greece; mariabozika29@gmail.com (M.B.); takaroschr@gmail.com (P.C.); elenipao@gmail.com (E.P.); chrispkyriazis@hotmail.com (C.K.); kassienmarie@gmail.com (K.-M.N.); gmpoliaris@gmail.com (G.B.); patrpan@hotmail.com (P.P.); angperperis@upatras.gr (A.P.); korosraf@hotmail.com (R.K.); iokoniari@yahoo.gr (I.K.); georgiaxiga@gmail.com (G.X.); efkapsali11@gmail.com (E.K.); alexandros.a.dedes@gmail.com (A.D.); anastaefrai@gmail.com (A.E.); dramoulias@live.com (A.M.); pdav@upatras.gr (P.D.); 2 Liverpool Centre for Cardiovascular Science, Liverpool L14 3PE, UK; 3 Department of Cardiology, Royal Brompton and Harefield Hospitals, Part of Guy’s and St Thomas’ NHS Foundation Trust, London SW3 6NP, UK; anastasisapostolos@gmail.com; 4 Faculty of Medicine, Imperial College London, London SW7 2AZ, UK; 5 Third Department of Cardiology, “Sotiria” Chest Diseases Hospital, School of Medicine, National and Kapodistrian University of Athens, 115 27 Athens, Greece; vbnikos@gmail.com

**Keywords:** left bundle branch area pacing, right ventricular pacing, myocardial work efficiency, pacing-induced cardiomyopathy, left ventricular ejection fraction, atrioventricular block

## Abstract

**Background/Objectives:** Evidence on myocardial-work indices beyond 12 months after left bundle branch area pacing (LBBAP) remains limited. We compared global myocardial work efficiency (GWE) and the left ventricular ejection fraction (LVEF) after LBBAP and right ventricular mid-septal pacing (RVP). **Methods:** This prospective, non-randomised cohort included 63 patients with advanced atrioventricular block and preserved pre-implantation LVEF: 34 assigned to an LBBAP strategy and 29 to RVP. Ventricular pacing burden was 100%. GWE was the primary endpoint; LVEF was the principal adjusted secondary endpoint. Analyses adjusted for age, sex, hypertension and atrial fibrillation. Follow-up averaged approximately two years, with an individual range of 0.64–4.23 years. **Results:** GWE was higher with LBBAP (85.23 ± 6.82% versus 80.00 ± 9.41%; *p* = 0.016), with an adjusted difference of +4.96 percentage points (95% CI 0.87 to 9.04; *p* = 0.018). The adjusted LVEF difference was +5.73 percentage points (95% CI 2.77 to 8.68; *p* < 0.001). Exploratory within-patient LVEF analyses showed mean changes of +1.20 percentage points with LBBAP (95% CI −1.25 to +3.65) and −6.15 with RVP (95% CI −9.16 to −3.14), with an unadjusted between-group difference of +7.35 percentage points (95% CI 3.55 to 11.16; *p* < 0.001). **Conclusions:** LBBAP was associated with higher GWE and LVEF at extended follow-up, although some propensity-score GWE confidence intervals included zero. LVEF-change findings are exploratory and do not estimate a common-time trajectory. These observational findings do not establish causality or clinical benefit.

## 1. Introduction

Left bundle branch area pacing (LBBAP) has emerged as an increasingly used alternative to right ventricular pacing (RVP), which can cause electrical and mechanical dyssynchrony [1], increase heart-failure hospitalisations, atrial fibrillation incidence, and pacing-induced cardiomyopathy (PICM) [2]. LBBAP engages the cardiac conduction system and achieves lower thresholds and better lead stability than His bundle pacing [3]. Besides cardiac resynchronisation therapy, LBBAP has been used to successfully treat bradyarrhythmias [4]. Additionally, LBBAP has been found to optimise electromechanical synchrony of the left ventricle (LV) [5] at the cost of interventricular dyssynchrony [6]. The recent European consensus paper regarding conduction-system pacing reflects the rapid expansion of the technique and provides contemporary advice on indications for His bundle pacing and LBBAP [7].

Although a recent registry has reported two-year outcomes comparing LBBAP with conventional pacing [8], data on myocardial-work indices remain limited to shorter follow-up periods, and no study has examined whether early differences in global myocardial work efficiency (GWE) persist beyond one year or are accompanied by later differences in left ventricular ejection fraction (LVEF). In this analysis, we present an extended follow-up of an expanded cohort building on our previously published study [9], with a mean follow-up of approximately two years and an individual range of 0.64–4.23 years.

## 2. Materials and Methods

### 2.1. Study Design

This is a prospective, non-randomised, single-centre cohort study conducted at the University Hospital of Patras, Greece. Patients were enrolled between July 2021 and November 2024; the echocardiographic assessments used in the present analysis were obtained between April 2022 and September 2026. The study was performed in accordance with the Declaration of Helsinki and approved by the institutional Scientific Board and Ethics Committee of the University Hospital of Patras (approval reference no. 234, 30 June 2021). All participants provided written informed consent. The 12-month outcomes of the original cohort have been previously reported [9]. The present analysis extends that the work by reporting outcomes of (i) the original 12-month cohort followed for an additional period and (ii) consecutive patients subsequently enrolled into the same protocol. Reporting follows the STROBE statement for observational studies.

### 2.2. Study Population

Eligibility criteria have been previously described [9]. Patients aged 18–90 years with second- or third-degree atrioventricular block, LVEF > 50%, an anticipated ventricular pacing burden ≥ 20%, and interventricular septum (IVS) thickness > 8 mm were eligible. Exclusion criteria included indication for cardiac resynchronisation therapy, defibrillation or leadless pacing; severe tricuspid regurgitation; and inability to implant from the left side.

### 2.3. Implantation Procedure

The pacing strategy was chosen by the operator after assessment of anatomical feasibility and patient-specific factors at implantation; these reasons for allocation were not recorded as study variables. No formal random allocation was used. Implantation was performed via ultrasound-guided transaxillary venous access using a micropuncture technique in both groups [10]. In the conventional pacing arm, right ventricular pacing was systematically targeted to the right ventricular mid-septum rather than the right ventricular apex. A manually shaped stylet directed the lead toward the septum, with orientation confirmed fluoroscopically in right and left anterior oblique projections; outflow tract and apical positions were not accepted. In the LBBAP arm, the Medtronic 3830 lumenless lead (Medtronic, Minneapolis, MN, USA) was deployed through a 315 His-dedicated sheath. Procedures were performed by two experienced operators (G.L. and A.P.), each with at least 100 LBBAP cases prior to enrollment of the present cohort. Throughout the manuscript, RVP refers to conventional right ventricular mid-septal pacing.

#### 2.3.1. Capture Criteria and Pacing Classification

Confirmation of left bundle branch capture followed the EHRA Consensus Statement [11]. Emphasis was placed on demonstrating transition during threshold testing or programmed extrastimuli rather than on the Stim-to-V6 R-wave peak time surrogate alone. Full capture criteria have been previously published [9]. Patients in whom transition could not be demonstrated despite confirmed deep septal lead position were classified as left ventricular septal pacing (LVSP).

The primary analysis classified patients according to the initial implantation strategy (attempted LBBAP versus RVP).

#### 2.3.2. Device Programming

All devices were programmed in the DDD(R) mode at a basic rate of 60 bpm. Paced and sensed atrioventricular delays were fixed at 150 and 120 ms, respectively. Ventricular pacing was set to unipolar mode. Algorithms minimising ventricular pacing were disabled in all patients.

### 2.4. Data Collection and Follow-Up

Preprocedural data included rhythm, pacing indication, QRS duration, echocardiographic parameters and laboratory parameters. Procedural data included procedure and fluoroscopy time, dose-area product (DAP), crossovers and post-procedural QRS duration. Periprocedural complications and length of post-procedural hospitalisation were documented (Supplementary Table S4). Clinically significant haematoma was defined by BRUISE CONTROL criteria [12]; all other haematomas were classified as non-significant.

Echocardiographic studies were performed using a Vivid E9 system (GE Healthcare, Milwaukee, WI, USA) with an M5S 3.5 MHz transducer. Conventional measurements (LVEF, left ventricular end-diastolic diameter [LVEDD], left ventricular end-systolic diameter [LVESD]) and global longitudinal strain (GLS) were obtained according to current guidelines [13,14]. Myocardial-work indices, global constructive work (GCW), global wasted work (GWW) and GWE, and peak strain dispersion (PSD) were derived using EchoPAC version 206 (GE Healthcare, Chicago, IL, USA). Myocardial work analysis followed established pressure–strain loop methodology [15]. Representative examples of two-dimensional speckle-tracking strain analysis used to derive GLS and PSD, and of myocardial work analysis used to derive global work index (GWI), GCW, GWW and GWE, are shown in Supplementary Figures S2 and S3. The methodology of myocardial work analysis has been previously described [9]. Device interrogation included pacing threshold, R-wave amplitude and impedance. The cardiologists could not be blinded to pacing modality, because echocardiographic features such as the course of the ventricular lead and the paced QRS morphology indirectly disclose treatment assignment; measurements were, however, performed according to a standardised protocol.

For reproducibility analysis, 35 randomly selected studies were re-analysed at one-week intervals. The original observer was blinded to the prior measurements (intra-observer), and the second observer was blinded to the first observer’s results (inter-observer).

### 2.5. Endpoints

GWE was the primary endpoint of the original study and was retained as the primary endpoint for the present extended follow-up analysis. LVEF is the principal adjusted secondary endpoint for this analysis; its *p* value is not corrected for multiplicity. All remaining comparisons, including GCW, GWW, the GWW/GCW ratio, GLS, PSD, linear dimensions, pacing and laboratory parameters, procedure and fluoroscopy time, dose-area product, and complications, are exploratory and were analysed without covariate adjustment or multiplicity correction. Their *p* values are interpreted as nominal, descriptive findings. Interpretation emphasises effect estimates and their uncertainty; 95% confidence intervals are reported for the primary adjusted comparison, adjusted LVEF and sensitivity analyses.

### 2.6. Statistical Analysis

The original sample-size calculation has been reported [9]. Briefly, assuming a GWE of 90.7% versus 80.8%, 14 patients per arm were required for 90% power at α = 0.05. No separate sample-size calculation was performed for the present extended follow-up analysis, which comprises 34 LBBAP and 29 RVP patients; the earlier calculation related to the original 12-month comparison and assumed a larger GWE difference than was observed here, so the precision of the present estimates is best reflected by the reported 95% confidence intervals. Baseline balance was assessed using standardised differences, with absolute values above 0.10 used as a descriptive indication of imbalance. Table 1 retains descriptive *p* values; Supplementary Table S8 reports standardised differences for baseline characteristics, implantation year and follow-up duration. Continuous variables are summarised as mean ± standard deviation or median [interquartile range], as specified in the tables. Distributional shape was assessed within each group using Shapiro–Wilk tests and normal Q-Q plots, considered together with the measurement scale and outliers rather than a Shapiro–Wilk threshold alone. Approximately normally distributed variables, including GWE, LVEF and GCW, were compared using Welch’s two-sample *t*-test, and skewed variables using the two-sided Mann–Whitney U test (for GWW and the GWW/GCW ratio, with a tie-adjusted normal approximation and continuity correction). Categorical variables are summarised as n (%) and compared using Fisher’s exact test.

The primary extended follow-up analyses included all 63 patients, for whom GWE, LVEF, pre-implantation LVEF and all primary-model covariates were available with no missing data. Adjusted analyses used an analysis of covariance (ANCOVA) on complete cases without imputation. The primary model included age, sex, hypertension, and atrial fibrillation: age and sex as established determinants of myocardial work; hypertension because non-invasive myocardial work incorporates a brachial cuff-derived pressure estimate; and atrial fibrillation because cycle-length variability affects strain and pressure–strain loop quality. To limit overfitting given the available sample size, adjustment was restricted to these four covariates. Six sensitivity ANCOVA models substituted, in turn, diabetes mellitus, dyslipidaemia, moderate tricuspid regurgitation, pacing indication, pre-implantation LVEF and follow-up duration for atrial fibrillation (Supplementary Table S7). Further sensitivity analyses added implantation year or implantation era to the primary model and restricted the cohort to defined follow-up windows. Implantation year was entered as a continuous calendar-year covariate; implantation era was defined as 2021–2022 versus 2023–2024, with 2021–2022 as the reference. To address treatment-selection bias more directly, a propensity score for assignment to an LBBAP strategy was estimated by logistic regression on age, sex, hypertension, atrial fibrillation, diabetes mellitus, dyslipidaemia, moderate tricuspid regurgitation, pacing indication and pre-implantation LVEF. The score was then used in four ways: as a single covariate, as quintile strata, as stabilised inverse-probability-of-treatment weights with heteroscedasticity-robust standard errors, and by restricting the cohort to the region of common support (Supplementary Table S9). Given the small sample and the number of covariates, these propensity-score estimates may be unstable and are reported as sensitivity analyses. Weights were capped at the pooled 99th percentile using R’s type-7 quantile (cap 4.86; one participant’s weight was reduced). Weighted-model standard errors used the heteroscedasticity-consistent type 3 (HC3) covariance estimator, with normal-Wald 95% confidence intervals and *p* values. Measured balance was assessed for each score covariate, before and after weighting, with Kish effective sample sizes (Supplementary Table S11). Standardised differences were the LBBAP-minus-RVP mean or proportion difference divided by the square root of the average within-group variance. For continuous covariates, unweighted differences used sample variances and weighted differences used population variances calculated as Σw(x − weighted mean)^2^/Σw; binary variances were p(1 − p), using weighted proportions after weighting. ANCOVA assumptions were assessed by inspection of residual and Q-Q plots. A further sensitivity analysis excluded the three LVSP patients and the one crossover. An additional post hoc sensitivity ANCOVA was performed after exclusion of the four RVP patients with hypothyroidism, in view of the recognised effect of thyroid status on myocardial function [16]. The rationale for covariate selection is detailed in the Supplementary Methods (Section S1). Full primary-adjustment model outputs are presented with the Results. Full diabetes-substituted and hypothyroidism-excluded model outputs are provided in Supplementary Tables S2 and S3. Hypothyroidism and former smoking each occurred in 0/34 LBBAP and 4/29 RVP patients; both were excluded from the primary adjustment set because their sparse, uneven distribution raised concerns about the precision of covariate adjustment. Their absence from one arm does not by itself make an additive linear model unidentified.

Pre-implantation LVEF was available for all participants. Exploratory within-group LVEF changes were assessed using paired *t*-tests and two-sided exact Wilcoxon signed-rank tests. Between-group differences in individual LVEF changes were assessed using Welch’s two-sample *t*-test and the Mann–Whitney U test. An ANCOVA of individual LVEF change additionally adjusted for age, sex, hypertension and atrial fibrillation (Supplementary Table S6). These analyses were exploratory and descriptive and concerned changes at each participant’s observed follow-up duration. No common-time longitudinal model was fitted. Pre-implantation myocardial-work indices were not obtainable, so the change in GWE from pre-implantation to extended follow-up could not be analysed. A repeated-measures analysis incorporating the 12-month assessment was not performed; published 12-month group summaries are used only for descriptive context. Multiplicity correction was not applied; global constructive work, global wasted work and their ratio are exploratory secondary endpoints, are reported without covariate adjustment, and do not provide confirmatory support for the primary endpoint. Intra- and inter-observer reproducibility were assessed using intraclass correlation coefficients (ICCs) with 95% confidence intervals from a two-way mixed-effects, absolute-agreement, single-measure model. All tests were two-sided with α = 0.05. Analyses were performed in R version 4.5.1 (R Foundation for Statistical Computing, Vienna, Austria).

## 3. Results

### 3.1. Patient Cohort and Baseline Characteristics

Baseline characteristics are presented in Table 1, and the patient flow diagram is shown in Supplementary Figure S1. Standardised differences indicate imbalance in several measured characteristics (Supplementary Table S8): 12 of the 30 displayed rows had absolute differences above 0.25 and 21 above 0.10. These rows include both categories of pacing indication, implantation year and follow-up duration. The largest differences were hypothyroidism and former smoking (both −0.57), dyslipidaemia (−0.47), moderate tricuspid regurgitation (−0.47), aspirin use (+0.46), pacing indication (±0.44), body mass index (+0.41) and beta-blocker use (−0.35). The baseline LVEF standardised difference was −0.31, despite its descriptive *p* value being above 0.05. Age (−0.05), atrial fibrillation (−0.06), intrinsic QRS duration (+0.07) and implantation year (+0.03) had smaller differences. Follow-up duration was 2.04 ± 0.94 years for LBBAP and 2.22 ± 0.92 years for RVP (median 1.77 years [interquartile range (IQR) 1.40–2.79; range 0.64–4.02] and 2.00 years [IQR 1.75–2.78; range 0.70–4.23], respectively; Welch’s *t*-test *p* = 0.445). Implantations were distributed across 2021, 2022, 2023 and 2024 with LBBAP shares of 54%, 50%, 60% and 50%, respectively; mean implantation year was 2022.44 ± 0.99 versus 2022.41 ± 1.02. Similar calendar-year distributions do not exclude confounding by operator choice or other unmeasured factors. The ventricular pacing burden was 100% in all patients.

### 3.2. Procedural Characteristics and Complications

Procedural characteristics are summarised in Supplementary Table S4. Total procedure time, fluoroscopy time and dose-area product were higher in the LBBAP group. At the final lead position, 30 of 34 patients in the LBBAP arm fulfilled left bundle branch capture criteria, three were classified as LVSP and one required crossover to RVP because of inability to penetrate the septum. One periprocedural interventricular septum perforation in the LBBAP group was corrected by lead repositioning without further sequelae. During follow-up, one lead displacement occurred in the RVP group and was managed without further complications. No pneumothorax, tamponade, clinically significant haematoma, or septal coronary artery damage was recorded in either group. Median post-procedural hospital stay was 2.30 days with LBBAP and 2.20 days with RVP (Mann–Whitney *p* = 0.849; Supplementary Table S4). No device infections were recorded during follow-up. Pacing parameters at extended follow-up are reported in Table 2; the study was not designed or powered to establish comparative long-term safety.

### 3.3. Twelve-Month Outcomes (Contextual Comparison)

Twelve-month outcomes for the original cohort have been reported previously [9]. Published 12-month group summaries are cited in the Discussion for descriptive context only and were not pooled with the present analysis.

### 3.4. Primary and Secondary Endpoints at Extended Follow-Up

Pacing, echocardiographic, laboratory and haemodynamic parameters are presented in Table 2. At the extended follow-up, GWE was higher in the LBBAP group than in the RVP group (85.23 ± 6.82% versus 80.00 ± 9.41%, Welch’s *t*-test *p* = 0.016). The adjusted difference was +4.96 percentage points (95% CI 0.87 to 9.04, *p* = 0.018); the full model is presented in Table 3. Across six sensitivity ANCOVA models substituting diabetes mellitus, dyslipidaemia, moderate tricuspid regurgitation, pacing indication, pre-implantation LVEF or follow-up duration for atrial fibrillation, adjusted GWE differences ranged from +4.45 to +4.92 percentage points (all nominal *p* ≤ 0.041; Supplementary Table S7). Adding the implantation year gave +4.97 percentage points (95% CI 0.87 to 9.07, *p* = 0.018), and adding the implantation era gave +4.91 percentage points (95% CI 0.79 to 9.03, *p* = 0.020). These exploratory analyses do not establish adequate confounding control.

GCW was higher in the LBBAP group (1809 ± 437 vs. 1582 ± 359 mmHg%, Welch *p* = 0.027). GWW did not differ significantly (median 246 [191.5–324.5] vs. 279 [220–505] mmHg%, Mann–Whitney *p* = 0.151), and the GWW/GCW ratio was lower in the LBBAP group (median 0.135 [0.110–0.186] vs. 0.206 [0.134–0.306], Mann–Whitney *p* = 0.046; Supplementary Table S5). These comparisons are exploratory, with nominal *p* values uncorrected for multiplicity, and do not provide confirmatory evidence for the primary GWE finding.

**Figure 1:**
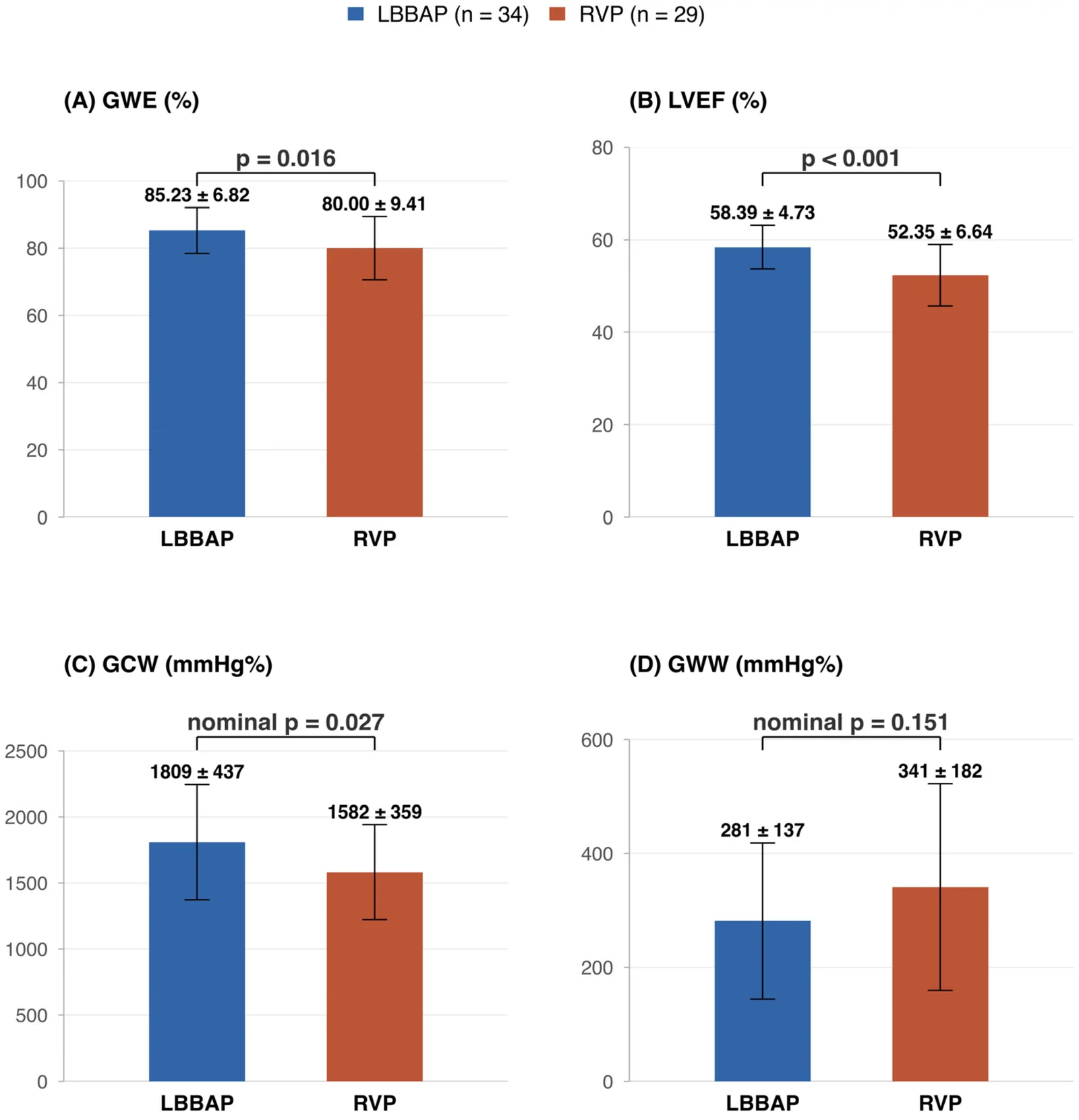
Myocardial work parameters and left ventricular ejection fraction at extended follow-up in the left bundle branch area pacing and right ventricular pacing groups. Bars represent observed mean ± standard deviation in the LBBAP (n = 34) and RVP (n = 29) groups. Displayed *p* values correspond to unadjusted between-group comparisons. GWE, LVEF and GCW *p* values are from Welch’s two-sample *t*-test; the GWW *p* value is from the Mann–Whitney U test. GCW and GWW are exploratory, and their displayed *p* values are nominal and uncorrected for multiplicity.

LVEF was higher in the LBBAP than RVP (58.39 ± 4.73% versus 52.35 ± 6.64%, Welch’s *t*-test *p* < 0.001). After ANCOVA adjustment, the difference was +5.73 percentage points (95% CI 2.77 to 8.68, *p* < 0.001); the full model is presented in Table 4. Pre-implantation LVEF showed no statistically detectable between-group difference (57.19 ± 4.46% versus 58.50 ± 4.12%, *p* = 0.230). In exploratory, descriptive paired analyses, mean within-patient LVEF change was +1.20 percentage points in LBBAP (95% CI −1.25 to +3.65; paired *t*-test *p* = 0.327) and −6.15 in RVP (95% CI −9.16 to −3.14; paired *t*-test *p* < 0.001). The between-group difference in change was +7.35 percentage points (95% CI 3.55 to 11.16; Welch *p* < 0.001), or +7.00 percentage points (95% CI 3.12 to 10.88, *p* < 0.001) after covariate adjustment (Supplementary Table S6). These change analyses describe observations at participants’ individual follow-up times and do not estimate a common-time longitudinal trajectory. Adding pre-implantation LVEF to the four primary-model covariates gave +5.57 percentage points (95% CI 2.57 to 8.57, *p* < 0.001) for LVEF and +5.05 percentage points (95% CI 0.88 to 9.21, *p* = 0.018) for GWE. Substituting pre-implantation LVEF for atrial fibrillation kept the model size constant and gave +5.61 percentage points (95% CI 2.64 to 8.58, *p* < 0.001) for LVEF and +4.79 percentage points (95% CI 0.59 to 8.99, *p* = 0.026) for GWE (Supplementary Table S7). A repeated-measures analysis incorporating the 12-month assessment was not performed. Published 12-month group summaries are used only for descriptive context and are not a paired comparison with the present extended assessment. Group summaries are shown in Figure 1.

### 3.5. Sensitivity Analysis

Because only 30 of the 34 patients assigned to an LBBAP strategy fulfilled definitive left bundle branch capture criteria, the sensitivity analysis excluded three LVSP patients and one crossover (30 LBBAP, 29 RVP; Table 5). GWE was 85.90 ± 6.58% versus 80.00 ± 9.41% (Welch *p* = 0.008); the adjusted difference was +5.50 percentage points (95% CI 1.35 to 9.65, *p* = 0.010). LVEF was 58.37 ± 4.72% versus 52.35 ± 6.64% (Welch *p* < 0.001); the adjusted difference was +5.74 percentage points (95% CI 2.69 to 8.79, *p* < 0.001).

Excluding four RVP patients with hypothyroidism retained 34 LBBAP and 25 RVP patients. The adjusted differences were +5.53 percentage points (95% CI 1.27 to 9.79, *p* = 0.012) for GWE and +5.60 percentage points (95% CI 2.44 to 8.76, *p* < 0.001) for LVEF; residual confounding by thyroid status cannot be excluded (Supplementary Table S3). Restricting follow-up to 12–36 months (n = 43) gave +3.96 percentage points (95% CI −0.56 to 8.48, *p* = 0.084) for GWE and +4.31 percentage points (95% CI 0.38 to 8.24, *p* = 0.032) for LVEF. In propensity-score analyses, GWE differences ranged from +3.69 to +5.33 percentage points. Quintile stratification gave an adjusted GWE difference of +4.15 percentage points (95% CI −0.69 to 9.00, *p* = 0.092), and common-support restriction gave +3.69 percentage points (95% CI −0.54 to 7.93, *p* = 0.086). Both confidence intervals included zero. The common-support analysis retained 57 patients: 28 LBBAP and 29 RVP, excluding six LBBAP and no RVP patients. Across the propensity-score analyses in Supplementary Table S9, estimated LVEF differences ranged from +4.78 to +6.53 percentage points, with all 95% confidence intervals above zero. Kish effective sample sizes after weighting were 21.9 for LBBAP and 19.9 for RVP; the pooled value was 41.7. The largest individual weights represented 13.7% and 14.3% of the respective group totals. Mean absolute standardised differences improved from 0.284 to 0.074, but age (+0.11) and baseline LVEF (+0.18) remained above 0.10; age imbalance increased (Supplementary Table S11). Estimated GWE differences were positive across these analyses, but their magnitudes and confidence intervals varied with the analytical approach and sample restriction.

### 3.6. Intra- and Inter-Observer Variability

Intra-observer ICCs ranged from 0.878 to 0.959 and inter-observer ICCs from 0.889 to 0.964 for myocardial-work indices (Supplementary Table S1).

## 4. Discussion

The main findings are: (1) at a mean follow-up exceeding two years, GWE was higher in the LBBAP group than in the RVP group (85.2 ± 6.8% vs. 80.0 ± 9.4%, *p* = 0.016); (2) LVEF was also higher in the LBBAP group (58.4 ± 4.7% vs. 52.3 ± 6.6%, *p* < 0.001); (3) both differences persisted after adjustment for age, sex, hypertension and atrial fibrillation; (4) exploratory, descriptive paired analyses showed a mean LVEF decline in the RVP group (−6.15 percentage points, 95% CI −9.16 to −3.14, *p* < 0.001) with no statistically detectable change in the LBBAP group (+1.20 percentage points, 95% CI −1.25 to +3.65), a difference in change of +7.35 percentage points (*p* < 0.001); and (5) the apparent enlargement of the LVEF difference relative to the previously reported 12-month timepoint remains descriptive, given the differing cohort sizes. Because allocation was not randomised, these findings should be interpreted as associative rather than causal. Estimated GWE differences favoured LBBAP across all reported analyses. However, 95% confidence intervals included zero in the propensity-score quintile, common-support and restricted follow-up-window analyses.

The comparator here was right ventricular mid-septal rather than apical pacing, which should be considered when comparing these findings with studies using apical RVP.

Recent studies provide context, although populations and pacing burdens differ. MELOS RELOADED [17], a propensity-matched registry of 3382 patients, associated LBBAP with lower four-year mortality (hazard ratio [HR] 0.53, 95% CI 0.42 to 0.65, *p* < 0.001). Bertini et al. [8] reported lower cardiovascular death or heart-failure hospitalisation at two years (6.3% versus 12.7%; reported HR 0.78, 95% CI 0.59 to 0.87). CSPACE [18] randomised 202 patients; conduction-system pacing reduced the composite of PICM, upgrade to biventricular pacing, heart-failure hospitalisation and mortality (HR 0.35, 95% CI 0.19 to 0.64, *p* < 0.001), predominantly through less PICM. González-Matos et al. [19] randomised 75 patients and analysed 70: the between-group difference in LVEF change was −5.8 percentage points (RVP minus conduction-system pacing; 95% CI −9.6 to −2.0, *p* = 0.0032), with more heart-failure admissions in RVP (22.6% versus 5.1%, *p* = 0.0302). Eligibility and pacing requirements varied. MELOS RELOADED required LVEF >40% and ventricular pacing >20%; our cohort required LVEF >50% and an anticipated pacing burden ≥20%, with observed ventricular pacing of 100%.

RVP induces ventricular dyssynchrony and diastolic dysfunction with adverse haemodynamic consequences. Jeong et al. [20], at a median follow-up of 31 months in patients with preserved ejection fraction, reported pacemaker-induced heart failure in 10%, particularly with pre-existing diastolic dysfunction (31% vs. 3%, *p* = 0.001).

LVEF is subject to inter- and intra-observer variability, and GLS, while more sensitive to subclinical dysfunction, is load-dependent. Of note, baseline LVEF was numerically higher in the RVP group than in the LBBAP group (58.5 ± 4.1% vs. 57.2 ± 4.5%, *p* = 0.230), whereas at extended follow-up LVEF was substantially higher in the LBBAP group (58.4 ± 4.7% vs. 52.3 ± 6.6%, *p* < 0.001). Exploratory, descriptive within-patient analysis showed a similar pattern: LVEF declined in the RVP group (−6.15 percentage points, 95% CI −9.16 to −3.14, *p* < 0.001), whereas in the LBBAP group no statistically detectable change was observed (+1.20 percentage points, 95% CI −1.25 to +3.65). The LBBAP interval spans a decline of 1.25 points and an improvement of 3.65 points and should not be read as establishing that LVEF was preserved; what the data support is that the change differed between groups (+7.35 percentage points, *p* < 0.001) and that the difference is driven principally by decline in the RVP arm. To address these limitations, we used myocardial work (MW), a less load-dependent index than strain alone, derived non-invasively from the LV pressure–strain relationship [21]. Because the method incorporates an estimated LV pressure curve, MW is not fully independent of loading conditions. The present analysis evaluates between-group differences in myocardial work at extended follow-up and cannot determine whether these differences precede LVEF changes. Our primary endpoint was GWE, which expresses the percentage of constructive myocardial work relative to the total work performed.

Manganaro et al. [22] reported normal GWE values of 95% in men and 96% in women. El Mahdiui et al. [23] reported a median GWE of 96% in healthy individuals, with lower values in post-ST-elevation myocardial infarction and heart failure with reduced ejection fraction patients, though not in those with cardiovascular risk factors alone (*p* = 0.59), consistent with Chan et al. [24]. These findings support the further investigation of GWE in patients undergoing ventricular pacing. The present study does not establish that lower GWE specifically reflects pacing-related myocardial dysfunction or validate its diagnostic or prognostic utility. Hypothyroidism was present in four RVP patients and none in the LBBAP group. Because thyroid status influences myocardial contractility and ejection fraction, a sensitivity ANCOVA excluding these patients was performed (Supplementary Table S3); adjusted differences in GWE and LVEF were materially unchanged. Residual confounding by thyroid status nonetheless remains possible.

In our expanded cohort, 30 of 34 patients fulfilled left bundle branch pacing capture criteria, three were classified as LVSP, and one required crossover to RVP. Pre-implantation GWE was not recorded because advanced atrioventricular block caused the loss in electromechanical synchrony and cycle-to-cycle variability. In the original 12-month cohort [9], GWE was significantly higher in the LBBAP group from the very first echocardiographic study post-implantation (91.5% vs. 85.6%, *p* = 0.003) and remained so at 12 months (90.8% vs. 85.8%, *p* = 0.011). At extended follow-up, GWE was also higher in the LBBAP group (85.2% vs. 80.0%, *p* = 0.016). These comparisons use published group summaries rather than a paired analysis of the original participants. They cannot establish within-patient GWE decline or attribute it to ageing or comorbidity.

The previously reported 12-month LVEF comparison (56.4% vs. 53.9%, *p* = 0.234) and the present extended follow-up comparison (58.4% vs. 52.3%, *p* < 0.001) arise from different cohort sizes and do not establish a treatment-by-time interaction. The observed between-group difference in LVEF at extended follow-up is compatible with published observations of pacing-related left ventricular dysfunction [25]; the present study cannot establish a biological sequence linking GWE differences to later LVEF changes, and longitudinal validation is required. Studies with mean follow-up exceeding two years remain limited, particularly those reporting myocardial-work indices. Wang et al. [26] studied patients aged ≥75 years (mean follow-up 31.0 ± 16.8 months). In the subgroup with ventricular pacing burden >20%, they reported a lower incidence of the composite outcome with LBBAP than RVP (15.1% vs. 21.1%, *p* = 0.036), with LVEF decreasing in the RVP group only. Okubo et al. [27] (mean follow-up 840 ± 369 days) showed that even with catheter-guided septal lead placement, right ventricular septal pacing patients more often developed the composite outcome (22.8% vs. 4.9%, *p* = 0.02). Our findings are consistent with these studies and add myocardial work data at extended follow-up.

Regarding GWE specifically, Chen et al. [28] found a significant difference after a 6-month follow-up (91.4 ± 5.0% vs. 80.9 ± 8.3%, *p* < 0.001) when ventricular pacing burden exceeded 40%. Our study differs in that all patients had 100% ventricular pacing and the mean follow-up was approximately two years, compared with six months in Chen et al. [28]. Mao et al. [29] showed that global MW was preserved in LBBAP but markedly decreased in RVP (2039 ± 369 vs. 1457 ± 537 mmHg%, *p* < 0.01) over 1 year.

Sharma et al. [30] demonstrated lower rates of heart-failure hospitalisation and all-cause mortality with LBBAP compared with RVP, particularly at pacing burdens >20%.

Pacing modality also affects GWE in His bundle pacing (HBP). Fehske et al. found higher GWE with HBP than RVP (90.7% vs. 80.8%, *p* = 0.0066) [31], at a shorter follow-up (4.2 ± 1.1 months). LBBAP and HBP produce similar acute haemodynamic improvement [32].

LVEF differed between groups while GLS did not show a statistically detectable difference (−16.9 ± 2.2% versus −16.4 ± 2.9%, *p* = 0.451). From the reported rounded group summaries, the estimated GLS difference was −0.50 percentage points, with an approximate 95% confidence interval from −1.82 to +0.82, compatible with no difference and with differences in either direction. The descriptive standardised difference for GLS was smaller in magnitude than for LVEF (standardised mean difference −0.19 versus +1.05; Supplementary Table S10). These measures describe different aspects of ventricular function, and their different *p* values alone do not establish discordant mechanisms or the incremental value of GWE. Non-significant comparisons for GLS, peak strain dispersion and linear dimensions should not be interpreted as evidence of equivalence. Precision is assessed from effect estimates and confidence intervals, rather than from power calculated using the observed effect sizes.

A physiological explanation is possible but remains hypothetical. GLS describes longitudinal shortening averaged across segments, whereas GWE incorporates an estimated LV pressure curve and the timing of shortening and lengthening. Pacing-related differences in ventricular activation could therefore affect these indices differently. The GWW/GCW comparison is exploratory and cannot provide confirmatory evidence for this mechanism. Peak strain dispersion also showed no statistically detectable between-group difference. The present data do not establish a mechanistic explanation for the pattern across imaging indices.

Loading conditions may contribute to the GWE difference. Brachial pressure supplies the pressure input to the non-invasive pressure–strain loop; systolic and diastolic pressure comparisons were not statistically significant (128 versus 122 mmHg, *p* = 0.434; 90 versus 87 mmHg, *p* = 0.410). These results do not demonstrate equivalent pressure distributions, exclude differences in loading conditions, or establish that the GWE difference arose solely from strain and its timing. Whether GWE provides incremental physiological or prognostic information beyond LVEF and GLS requires a study designed to assess that question.

Regarding QRS duration, in our study, paced QRS duration was significantly shorter in the LBBAP arm (122 ± 18 ms vs. 156 ± 19 ms, *p* < 0.001), consistent with Yang et al. [33] and Cai et al. [5], reflecting rapid recruitment of the LV His–Purkinje system during LBBAP.

Observed procedural and post-procedural complications included one interventricular septal perforation in the LBBAP arm and one lead displacement in the RVP arm (Supplementary Table S4). The study was neither designed nor powered for comparative safety assessment, so no conclusion about long-term comparative safety can be drawn. Pacing parameters at extended follow-up are reported in Table 2. Procedure and fluoroscopy times were higher in the LBBAP arm, consistent with the greater procedural complexity of this technique; the present data cannot isolate the contribution of operator learning.

### 4.1. Future Perspectives

Randomised controlled trials including OptimPacing, LEFT-HF and LEAP were designed to examine LBBAP versus RVP with hard clinical endpoints. Future multicentre studies with prespecified longitudinal imaging and clinical endpoints should test whether early myocardial-work differences predict later LVEF decline and clinical events. The present study cannot establish temporal precedence or validate GWE as a surrogate endpoint.

### 4.2. Limitations

This study has several limitations. First, it is a single-centre, non-randomised study with a small sample (N = 63). The pacing strategy was chosen using anatomical feasibility and patient-specific factors that were not recorded as study variables, so selection bias and confounding by indication remain possible. Standardised differences show imbalance in several measured characteristics (Supplementary Table S8). Across the six covariate-substitution models, the GWE estimate differed from the primary estimate by at most 0.51 percentage points (Supplementary Table S7); this consistency does not establish that the adjustment set is sufficient or exclude residual confounding. Propensity-score analyses produced positive GWE estimates, but the common-support estimate was smaller and two confidence intervals included zero (Supplementary Table S9). Sparse covariates, limited overlap, weight variability and the small sample limit these analyses. Second, pre-implantation LVEF was available for all 63 patients, permitting within-patient change analysis; pre-implantation myocardial work was not obtainable. Change in GWE from pre-implantation to extended follow-up cannot therefore be assessed. The present analysis does not determine whether GWE differences precede or predict LVEF decline. Third, the cardiologists could not be blinded to pacing modality because the lead course and paced QRS morphology disclosed assignment, creating potential measurement bias despite a standardised protocol. Fourth, clinical functional outcomes and heart-failure events were not systematically adjudicated; the imaging associations cannot establish clinical benefit or comparative safety. Fifth, within-patient LVEF-change analyses were exploratory and descriptive: follow-up duration varied from 0.64 to 4.23 years, and no common-time longitudinal model was fitted. A paired comparison incorporating the 12-month assessment was not performed. Published 12-month group summaries are used only for descriptive context. Duration adjustment and restricted-window analyses address this variation incompletely; the restricted samples had wider intervals and somewhat smaller estimates. Sixth, non-significant comparisons, including GLS, peak strain dispersion and linear dimensions, cannot establish equivalence. Exploratory endpoint comparisons and propensity-score sensitivity analyses have *p* values uncorrected for multiplicity. Finally, no separate sample-size calculation was performed for the extended analysis. The original calculation related to the 12-month comparison and assumed a larger GWE difference than observed here; uncertainty is best reflected by the reported confidence intervals.

## 5. Conclusions

At extended follow-up, LBBAP was associated with higher GWE and LVEF than RVP in the primary adjusted analyses. Estimated GWE differences favoured LBBAP across all reported analyses. However, 95% confidence intervals included zero in the propensity-score quintile, common-support and restricted follow-up-window analyses. Exploratory analyses of paired LVEF changes suggested a between-group difference at the observed follow-up times; they do not establish a common-time longitudinal trajectory. The present study cannot determine whether myocardial-work differences precede or predict LVEF decline or translate into clinical benefit. Validation in larger randomised studies with prespecified longitudinal imaging and clinical endpoints is required.

## Figures and Tables

**Table 1:** Baseline characteristics according to pacing strategy.

Characteristic	LBBAP (n = 34)	RVP (n = 29)	*p* Value
**Demographics**			
Age (years)	76.1 ± 8.2	76.6 ± 10.5	0.851
Male, n (%)	20 (59)	20 (69)	0.442
BMI (kg/m^2^)	28.7 ± 3.8	27.1 ± 4.1	0.116
**Comorbidities**			
Hypertension, n (%)	15 (44)	16 (55)	0.453
Dyslipidaemia, n (%)	11 (32)	16 (55)	0.080
Diabetes, n (%)	10 (29)	13 (45)	0.294
CAD, n (%)	1 (3)	1 (3)	1.000
Heart failure, n (%)	1 (3)	1 (3)	1.000
PAD, n (%)	7 (21)	3 (10)	0.319
CKD, n (%)	2 (6)	1 (3)	1.000
COPD, n (%)	2 (6)	3 (10)	0.654
Hypothyroidism, n (%)	0 (0)	4 (14)	0.040
Atrial fibrillation, n (%)	4 (12)	4 (14)	1.000
Stroke/TIA, n (%)	1 (3)	1 (3)	1.000
**Smoking**			
Current smoker, n (%)	2 (6)	1 (3)	1.000
Former smoker, n (%)	0 (0)	4 (14)	0.040
**Medications**			
Beta-blocker, n (%)	14 (41)	17 (59)	0.210
ACEi/ARB, n (%)	12 (35)	12 (41)	0.795
Anticoagulants, n (%)	2 (6)	3 (10)	0.654
ASA, n (%)	14 (41)	6 (21)	0.107
P2Y12 inhibitor, n (%)	7 (21)	6 (21)	1.000
**Pacing indication and electrocardiography**			
2nd degree AVB, n (%)	19 (56)	10 (34)	0.129
3rd degree AVB, n (%)	15 (44)	19 (66)	0.129
Intrinsic QRS duration (ms)	112.4 ± 25.8	110.7 ± 24.3	0.789
**Echocardiography**			
LVEF (%)	57.19 ± 4.46	58.50 ± 4.12	0.230
**Tricuspid regurgitation**			
None/mild, n (%)	29 (85)	19 (66)	0.081
Moderate, n (%)	5 (15)	10 (34)	
**Laboratory**			
eGFR (mL/min/1.73 m^2^)	56.2 ± 25.4	58.3 ± 22.8	0.731
Creatinine (mg/dL)	1.3 ± 1.2	1.2 ± 0.6	0.756

Continuous values are mean ± standard deviation. Categorical variables are shown as n (%). *p* values were obtained using Welch’s two-sample *t*-test for normally distributed continuous variables, the Mann–Whitney U test for skewed continuous variables, and Fisher’s exact test for categorical variables. ACEi, angiotensin-converting enzyme inhibitor; ARB, angiotensin receptor blocker; ASA, acetylsalicylic acid; AVB, atrioventricular block; BMI, body mass index; CAD, coronary artery disease; CKD, chronic kidney disease; COPD, chronic obstructive pulmonary disease; eGFR, estimated glomerular filtration rate; LBBAP, left bundle branch area pacing; LVEF, left ventricular ejection fraction; PAD, peripheral artery disease; QRS, QRS-complex duration; RVP, right ventricular mid-septal pacing; TIA, transient ischaemic attack. Baseline *p* values are descriptive. The tricuspid-regurgitation *p* value compares the complete two-category distribution.

**Table 2:** Pacing, echocardiographic, laboratory and haemodynamic parameters at extended follow-up.

Characteristic	LBBAP (n = 34)	RVP (n = 29)	*p* Value
**Pacing parameters**			
Paced QRS duration (ms)	122 ± 18	156 ± 19	<0.001
Pacing threshold (V)	0.7 ± 0.2	0.7 ± 0.3	0.469
Sensing amplitude (mV)	11.0 ± 3.0	11.6 ± 2.1	0.370
Impedance (Ω)	468 ± 81	552 ± 95	<0.001
V6 RWAT (ms)	66 [58.9–67.1]	N/A	—
V6–V1 (ms)	43.0 [39.2–46.9]	N/A	—
Atrial pacing percentage (%)	34.3 [20.0–62.1]	25.4 [18.1–53.4]	—
Ventricular pacing percentage (%)	100	100	—
**Laboratory**			
NT-proBNP (pg/mL)	343 [271–435]	393 [324–478]	0.167
**Echocardiographic endpoints**			
LVEF (%)	58.39 ± 4.73	52.35 ± 6.64	<0.001
LVEDD (mm)	48.1 ± 5.53	48.9 ± 5.49	0.564
LVESD (mm)	34.8 ± 5.31	35.2 ± 5.51	0.771
GLS (%)	−16.9 ± 2.2	−16.4 ± 2.9	0.451
**Myocardial work parameters**			
GCW (mmHg%)	1809 ± 437	1582 ± 359	0.027
GWW (mmHg%)	246 [191.5–324.5]	279 [220–505]	0.151
GWE (%)	85.23 ± 6.82	80.00 ± 9.41	0.016
PSD (ms)	61.9 ± 15.23	63.5 ± 18.3	0.710
**Hemodynamics**			
Systolic BP (mmHg)	128 [110–161]	122 [120–151]	0.434
Diastolic BP (mmHg)	90 [75–92]	87 [73–89]	0.410

Values are mean ± standard deviation, except where indicated by square brackets, which denote median [interquartile range]. *p* values were obtained using Welch’s two-sample *t*-test for normally distributed continuous variables and the Mann–Whitney U test for skewed continuous variables. BP, blood pressure; GCW, global constructive work; GLS, global longitudinal strain; GWE, global myocardial work efficiency; GWW, global wasted work; LBBAP, left bundle branch area pacing; LVEDD, left ventricular end-diastolic diameter; LVEF, left ventricular ejection fraction; LVESD, left ventricular end-systolic diameter; N/A, not applicable; NT-proBNP, N-terminal pro-B-type natriuretic peptide; PSD, peak strain dispersion; QRS, QRS-complex duration; RVP, right ventricular mid-septal pacing; RWAT, R-wave peak time. GWE, LVEF and GCW were compared using Welch’s two-sample *t*-test. GWW was compared using the two-sided Mann–Whitney U test with a tie-adjusted normal approximation and continuity correction. GCW and GWW comparisons are exploratory; their *p* values are nominal and uncorrected for multiplicity.

**Table 3:** ANCOVA for GWE at extended follow-up.

Term	Estimate (β)	95% CI	*p* Value	Partial η^2^
Intercept	+82.811	64.76 to 100.86	—	—
LBBAP (vs. RVP)	+4.956	0.87 to 9.04	0.018	0.094
Age (yr)	−0.016	−0.25 to 0.22	0.893	0.000
Sex (male)	−2.468	−6.88 to 1.95	0.268	0.021
Hypertension	−1.057	−5.55 to 3.44	0.640	0.004
Atrial fibrillation	+4.913	−1.54 to 11.36	0.133	0.039

Outcome, GWE (%). Overall model F(5, 57) = 2.58, *p* = 0.036; R^2^ = 0.184; adjusted R^2^ = 0.113; n = 63 (residual df = 57). Adjusted LBBAP-minus-RVP difference +4.96 percentage points (95% CI 0.87 to 9.04, *p* = 0.018). Models include pacing strategy, age, sex, hypertension and atrial fibrillation. β is an unstandardised coefficient in percentage points; the age coefficient is per year. Reference categories are RVP, female sex, and absence of the binary condition. Each non-intercept term has numerator df = 1; partial η^2^ = t^2^/(t^2^ + residual df). Confidence intervals and *p* values use the Student t distribution. An em dash denotes an intercept statistic not reported. ANCOVA, analysis of covariance; CI, confidence interval; df, degrees of freedom; GWE, global myocardial work efficiency; LBBAP, left bundle branch area pacing; RVP, right ventricular mid-septal pacing.

**Table 4:** ANCOVA for LVEF at extended follow-up.

Term	Estimate (β)	95% CI	*p* Value	Partial η^2^
Intercept	+52.862	39.80 to 65.92	—	—
LBBAP (vs. RVP)	+5.727	2.77 to 8.68	<0.001	0.209
Age (yr)	+0.019	−0.15 to 0.19	0.821	0.001
Sex (male)	−1.902	−5.10 to 1.29	0.238	0.024
Hypertension	−1.052	−4.31 to 2.20	0.520	0.007
Atrial fibrillation	−0.677	−5.34 to 3.99	0.772	0.001

Outcome, LVEF (%). Overall model F(5, 57) = 3.90, *p* = 0.004; R^2^ = 0.255; adjusted R^2^ = 0.189; n = 63 (residual df = 57). Adjusted LBBAP-minus-RVP difference +5.73 percentage points (95% CI 2.77 to 8.68, *p* < 0.001). Models include pacing strategy, age, sex, hypertension and atrial fibrillation. β is an unstandardised coefficient in percentage points; the age coefficient is per year. Reference categories are RVP, female sex, and absence of the binary condition. Each non-intercept term has numerator df = 1; partial η^2^ = t^2^/(t^2^ + residual df). Confidence intervals and *p* values use the Student t distribution. An em dash denotes an intercept statistic not reported. ANCOVA, analysis of covariance; CI, confidence interval; df, degrees of freedom; LBBAP, left bundle branch area pacing; LVEF, left ventricular ejection fraction; RVP, right ventricular mid-septal pacing.

**Table 5:** Capture-confirmed sensitivity analysis of GWE and LVEF at extended follow-up.

Outcome	LBBAP (n = 30)	RVP (n = 29)	Unadjusted *p* Value	Adjusted Mean Difference (95% CI)	Adjusted *p* Value
GWE (%)	85.90 ± 6.58	80.00 ± 9.41	0.008	+5.50 (1.35 to 9.65)	0.010
LVEF (%)	58.37 ± 4.72	52.35 ± 6.64	<0.001	+5.74 (2.69 to 8.79)	<0.001

Values are mean ± standard deviation. The sensitivity analysis excluded three patients classified as left ventricular septal pacing and one crossover from attempted LBBAP to RVP (n = 59). Unadjusted *p* values use Welch’s two-sample *t*-test for both outcomes. Shapiro–Wilk tests did not detect departures from normality in either group (LBBAP/RVP: GWE *p* = 0.84/0.96; LVEF *p* = 0.98/0.44). Adjusted differences are in percentage points, LBBAP minus RVP, from ANCOVA with age, sex, hypertension and atrial fibrillation; 95% CIs and *p* values use the Student t distribution (residual df = 53). These sensitivity *p* values are nominal and uncorrected for multiplicity. ANCOVA, analysis of covariance; CI, confidence interval; df, degrees of freedom; GWE, global myocardial work efficiency; LBBAP, left bundle branch area pacing; LVEF, left ventricular ejection fraction; RVP, right ventricular mid-septal pacing.

## Data Availability

The raw data supporting the conclusions of this article will be made available by the authors on request.

## Supplementary Materials

The following supporting information can be downloaded at: https://www.mdpi.com/article/10.3390/jcm15218224/s1. Section S1: Supplementary Methods; Table S1: Intra- and inter-observer reproducibility of myocardial-work indices; Table S2: Sensitivity ANCOVA with diabetes mellitus replacing atrial fibrillation; Table S3: Sensitivity ANCOVA excluding RVP patients with hypothyroidism; Table S4: Procedural characteristics and recorded complications; Table S5: Myocardial-work indices and LVEF at extended follow-up; Table S6: Within-patient LVEF change from pre-implantation to extended follow-up; Table S7: Sensitivity analyses for alternative covariates, calendar time and follow-up duration; Table S8: Standardised differences in participant characteristics, implantation year and follow-up duration; Table S9: Propensity-score sensitivity analyses of GWE and LVEF; Table S10: Descriptive standardised mean differences for echocardiographic endpoints; Table S11: Propensity-score coefficients and covariate balance before and after weighting; Figure S1: Patient flow diagram; Figure S2: Representative two-dimensional speckle-tracking longitudinal strain analysis; Figure S3: Representative myocardial work analysis.

## Abbreviations

The following abbreviations are used in this manuscript:

ANCOVA	Analysis of covariance
AVB	Atrioventricular block
DAP	Dose-area product
GCW	Global constructive work
GLS	Global longitudinal strain
GWE	Global myocardial work efficiency
GWI	Global work index
GWW	Global wasted work
HBP	His bundle pacing
ICC	Intraclass correlation coefficient
IVS	Interventricular septum
LBBAP	Left bundle branch area pacing
LV	Left ventricle/left ventricular
LVEDD	Left ventricular end-diastolic diameter
LVEF	Left ventricular ejection fraction
LVESD	Left ventricular end-systolic diameter
LVSP	Left ventricular septal pacing
MW	Myocardial work
PICM	Pacing-induced cardiomyopathy
PSD	Peak strain dispersion
RVP	Right ventricular (mid-septal) pacing
