# Supplementary material for: Global Myocardial Work Efficiency and Left Ventricular Ejection Fraction After Left Bundle Branch Area Pacing Versus Right Ventricular Pacing: Extended Follow-Up of a Prospective Cohort
Source: J Clin Med. 2026 Oct 22;15(21):8224. doi: 10.3390/jcm15218224 (PMC13764148; doi:10.3390/jcm15218224)
Supplement: Supplementary file 1 [file jcm-15-08224-s001.zip › jcm-4621297-supplementary.pdf]

Supplementary Materials

Supplementary materials for the manuscript: Global myocardial work efficiency and left ventricular ejection fraction after left bundle branch area pacing versus right ventricular pacing: extended follow-up of a prospective cohort.

Correspondence: Georgios Leventopoulos, MD, PhD; Department of Medicine, Division of Cardiology, University Hospital of Patras, 265 04 Patras, Greece; Email: levent2669@gmail.com.

Section S1. Supplementary Methods

Adjusted analyses

Adjusted comparisons of the primary endpoint and principal adjusted secondary endpoint, global myocardial work efficiency (GWE) and left ventricular ejection fraction (LVEF), at extended follow-up were performed with analysis of covariance (ANCOVA). Each model included the pacing strategy (left bundle branch area pacing [LBBAP] vs. right ventricular pacing [RVP]) as the factor of interest and four covariates selected for their pathophysiological relevance to myocardial work and their potential to confound treatment allocation in a non-randomised cohort: age and sex as established determinants of myocardial work; hypertension because non-invasive myocardial work incorporates a brachial cuff-derived pressure estimate; and atrial fibrillation because cycle-length variability affects strain and pressure-strain loop quality. Models were fitted on complete cases; no imputation was performed. Model assumptions were assessed by inspection of residual and normal quantile-quantile plots. The covariates were included for confounding control rather than for prediction, so the overall model F test and R^2 are not the quantities of interest; inference concerns the adjusted pacing-strategy term, which is reported with its 95% confidence interval throughout.

Sensitivity analyses

Sensitivity analyses included several approaches. In the first, atrial fibrillation was replaced in turn by diabetes mellitus (Table S2), dyslipidaemia, moderate tricuspid regurgitation, pacing indication, pre-implantation LVEF and follow-up duration, giving six parsimonious models each addressing one alternative confounding structure (Table S7). Further analyses in this group added implantation year or implantation era to the primary model, and restricted the cohort to defined follow-up windows (Table S7). In the second, the three patients classified as left ventricular septal pacing (LVSP) and the one crossover from attempted LBBAP to RVP were excluded, leaving 30 LBBAP and 29 RVP patients (main-text Table 5). In a post hoc analysis, the four RVP patients with hypothyroidism were excluded, given the recognised effect of thyroid status on myocardial function, leaving 34 LBBAP and 25 RVP patients (59 total; Table S3). Hypothyroidism and former smoking, each present in 4 of 29 RVP patients and in none of the 34 LBBAP patients, were not entered as covariates because their sparse and uneven

distribution raised concerns about the precision of adjustment.

Reporting and reproducibility

Adjusted between-group differences are reported as unstandardised regression coefficients (β) with 95% confidence intervals and two-sided p values; partial η^2 is reported for each model term. GCW was compared using Welch's two-sample t-test; GWW and GWW/GCW were compared using two-sided Mann-Whitney U tests with tie-adjusted normal approximations and continuity correction. Intra- and inter-observer reproducibility of myocardial work indices was assessed in 35 randomly selected studies with intraclass correlation coefficients from a two-way mixed-effects, absolute-agreement, single-measure model, with 95% confidence intervals (Table S1). Analyses were performed in R, version 4.5.1; all tests were two-sided at the 0.05 level.

Analytic sample size

Sixty-three patients were enrolled (34 LBBAP and 29 RVP), with complete data on both outcomes, on pre-implantation LVEF and on all four covariates; no imputation was required. Because pre-implantation LVEF was available for every participant, exploratory, descriptive analyses of within-patient LVEF change were performed using paired t-test and two-sided exact Wilcoxon signed-rank test within each group and compared between groups (Table S6); the between-group comparison concerns paired changes at each participant's observed follow-up duration, rather than a common-time trajectory. Pre-implantation myocardial work indices were not obtainable, because atrioventricular block rendered pressure-strain analysis non-interpretable, so change in GWE from pre-implantation to extended follow-up could not be analysed. The primary-adjustment GWE and LVEF models and both diabetes-substituted models therefore included all 63 participants. The two hypothyroidism-excluded models included the 59 patients remaining after exclusion of the 4 RVP patients with hypothyroidism. The footnotes to main Tables 3 and 4 and Supplementary Tables S2 and S3 report the analytic sample and model degrees of freedom.

Additional analyses

Standardised differences were used to describe measured balance (Table S8). Propensity-score sensitivity analyses used a logistic model for initial LBBAP assignment with age, sex, hypertension, atrial fibrillation, diabetes, dyslipidaemia, moderate tricuspid regurgitation, pacing indication and pre-implantation LVEF. The score was used as a covariate, as quintile strata, for stabilised inverse-probability weighting with HC3 heteroscedasticity-consistent standard errors and normal-Wald confidence intervals and p values, and to restrict to common support (Table S9). Each approach had its own outcome model: outcome on pacing strategy plus the score; outcome on pacing strategy plus stratum indicators; a weighted model of outcome on pacing strategy; and the primary covariate model refitted after restriction. Stabilised weights were $P(T=1)/e$ for treated and $P(T=0)/(1-e)$ for control participants, capped at the pooled 99th percentile using R's type-7 sample quantile. Covariate balance was assessed with standardised differences for each covariate individually, before and after weighting, together with Kish effective sample sizes (Table S11). These analyses are exploratory and

cannot remove confounding by unmeasured factors. Table S10 reports group summaries and descriptive standardised effect sizes. Post-hoc power based on observed effects is not used to interpret nonsignificant findings.

Section S2. Supplementary Tables

Table S1. Intra- and interobserver reproducibility of myocardial-work indices.

Variables	Intraobserver variability		Interobserver variability	
	ICC	95% CI	ICC	95% CI
GWI (mmHg%)	0.954	0.911-0.976	0.919	0.847-0.958
GCW (mmHg%)	0.959	0.921-0.979	0.964	0.930-0.982
GWW (mmHg%)	0.878	0.773-0.936	0.920	0.848-0.959
GWE (%)	0.949	0.902-0.974	0.889	0.793-0.942

ICCs were calculated from 35 studies using a two-way mixed-effects, absolute-agreement, single-measure model; 95% CIs are shown. CI, confidence interval; GCW, global constructive work; GWE, global myocardial work efficiency; GWI, global work index; GWW, global wasted work; ICC, intraclass correlation coefficient.

Table S2. Sensitivity ANCOVA with diabetes mellitus replacing atrial fibrillation.

GWE model

Term	Estimate (β)	95% CI	p value	Partial η²
LBBAP (vs RVP)	+4.917	0.72 to 9.11	0.022	0.088
Age (yr)	−0.030	−0.27 to 0.21	0.801	0.001
Sex (male)	−2.705	−7.23 to 1.82	0.236	0.025
Hypertension	−2.050	−6.70 to 2.60	0.381	0.014
Diabetes mellitus	+1.304	−3.22 to 5.83	0.566	0.006

Outcome, GWE (%). Overall model $F(5, 57) = 2.11$, $p = 0.078$; $R^2 = 0.156$; adjusted $R^2 = 0.082$; n = 63 (residual df = 57). Adjusted LBBAP-minus-RVP difference +4.92 percentage points (95% CI 0.72 to 9.11, $p = 0.022$).

.

LVEF model

Term	Estimate (β)	95% CI	p value	Partial η²
LBBAP (vs RVP)	+6.051	3.10 to 9.00	<0.001	0.229
Age (yr)	+0.043	−0.13 to 0.21	0.610	0.005
Sex (male)	−1.476	−4.65 to 1.70	0.356	0.015
Hypertension	−1.482	−4.74 to 1.78	0.367	0.014
Diabetes mellitus	+1.975	−1.20 to 5.15	0.218	0.026

Outcome, LVEF (%). Overall model $F(5, 57) = 4.29$, $p = 0.002$; $R^2 = 0.273$; adjusted $R^2 = 0.210$; n = 63 (residual df = 57). Adjusted LBBAP-minus-RVP difference +6.05 percentage points (95% CI 3.10 to 9.00, $p < 0.001$). Models include pacing strategy, age, sex, hypertension and diabetes mellitus. β is an unstandardised coefficient in percentage points; the age coefficient is per year. Reference categories are RVP, female sex, and absence of the binary condition. Each non-intercept term has numerator df = 1; partial $\eta^2 = t^2/(t^2 + \text{residual df})$. Confidence intervals and p values use the Student t distribution. ANCOVA, analysis of covariance; CI, confidence interval; df, degrees of freedom; GWE, global myocardial work efficiency; LBBAP, left bundle branch area pacing; LVEF, left ventricular ejection fraction; RVP, right ventricular mid-septal pacing.

Table S3. Sensitivity ANCOVA excluding RVP patients with hypothyroidism.

GWE model

Term	Estimate (β)	95% CI	p value	Partial η²
LBBAP (vs RVP)	+5.530	1.27 to 9.79	0.012	0.114
Age (yr)	−0.051	−0.29 to 0.19	0.675	0.003
Sex (male)	−2.062	−6.57 to 2.44	0.363	0.016
Hypertension	−0.872	−5.51 to 3.77	0.708	0.003
Atrial fibrillation	+2.706	−4.87 to 10.28	0.477	0.010

Outcome, GWE (%). Overall model $F(5, 53) = 2.17$, $p = 0.071$; $R^2 = 0.170$; adjusted $R^2 = 0.092$; n = 59 (residual df = 53). Adjusted LBBAP-minus-RVP difference +5.53 percentage points (95% CI 1.27 to 9.79, $p = 0.012$).

LVEF model

Term	Estimate (β)	95% CI	p value	Partial η^2
LBBAP (vs RVP)	+5.600	2.44 to 8.76	<0.001	0.192
Age (yr)	+0.032	−0.15 to 0.21	0.720	0.002
Sex (male)	−1.878	−5.23 to 1.47	0.266	0.023
Hypertension	−0.973	−4.42 to 2.47	0.574	0.006
Atrial fibrillation	+0.240	−5.39 to 5.87	0.932	0.000

Outcome, LVEF (%). Overall model $F(5, 53) = 3.21$, $p = 0.013$; $R^2 = 0.232$; adjusted $R^2 = 0.160$; $n = 59$ (residual df = 53). Adjusted LBBAP-minus-RVP difference +5.60 percentage points (95% CI 2.44 to 8.76, $p < 0.001$). Models include pacing strategy, age, sex, hypertension and atrial fibrillation. β is an unstandardised coefficient in percentage points; the age coefficient is per year. Reference categories are RVP, female sex, and absence of the binary condition. Each non-intercept term has numerator df = 1; partial $\eta^2 = t^2/(t^2 + \text{residual df})$. Confidence intervals and p values use the Student t distribution. ANCOVA, analysis of covariance; CI, confidence interval; df, degrees of freedom; GWE, global myocardial work efficiency; LBBAP, left bundle branch area pacing; LVEF, left ventricular ejection fraction; RVP, right ventricular mid-septal pacing. After exclusion of four RVP patients with hypothyroidism, the adjusted GWE difference was +5.53 versus +4.96 percentage points in the primary model; the LVEF difference was +5.60 versus +5.73. Residual confounding by thyroid status cannot be excluded.

Table S4. Procedural characteristics and recorded complications.

Characteristic	LBBAP (n = 34)	RVP (n = 29)	p value
Total time (min)	89 [69-100]	50 [40-55]	<0.001
Fluoroscopy time (sec)	700 [523-1001]	102 [70-150]	<0.001
DAP (cGy·cm²)	889 [498-1120]	260 [130-396]	<0.001
Crossover, n (%)	1 (3)	0 (0)	1.000
Pneumothorax, n (%)	0 (0)	0 (0)	-
Tamponade, n (%)	0 (0)	0 (0)	-
IVS perforation, n (%)	1 (3)	0 (0)	1.000
AMI (septal perforator), n (%)	0 (0)	0 (0)	-
Lead displacement, n (%)	0 (0)	1 (3)	0.460
Clinically significant haematoma, n (%)	0 (0)	0 (0)	-
Non-significant haematoma, n (%)	2 (6)	3 (10)	0.654
Fever, n (%)	5 (15)	3 (10)	0.716
Antibiotics, n (%)	5 (15)	5 (17)	1.000
In-hospital stay (days)	2.30 [1.89-3.00]	2.20 [1.95-3.00]	0.849

Values are median [interquartile range] or n (%). AMI, acute myocardial infarction; DAP, dose-area product; IQR, interquartile range; IVS, interventricular septum; LBBAP, left bundle branch area pacing; RVP, right ventricular mid-septal pacing. Continuous variables were compared using the Mann–Whitney U test and categorical variables using Fisher's exact test.

Table S5. Myocardial-work indices and LVEF at extended follow-up.

**Parameter**	**LBBAP (n = 34)**	**RVP (n = 29)**	Unadjusted p value	**Adjusted effect (95% CI)**	Adjusted p value
GWE (%)	85.23 ± 6.82	80.00 ± 9.41	0.016	+4.96 (0.87 to 9.04)	0.018
GCW, mmHg%	1809 ± 437	1582 ± 359	0.027	-	-
GWW, mmHg%	246 [191.5–324.5]	279 [220–505]	0.151	-	-
GWW/GCW ratio	0.135 [0.110–0.186]	0.206 [0.134–0.306]	0.046	-	-
LVEF (%)	58.39 ± 4.73	52.35 ± 6.64	<0.001	+5.73 (2.77 to 8.68)	<0.001

Values are mean ± standard deviation or median [interquartile range], as indicated. Adjusted GWE and LVEF comparisons used analysis of covariance (ANCOVA) with age, sex, hypertension and atrial fibrillation. Unadjusted GWE, LVEF and GCW comparisons used Welch's two-sample t-test. GWW and GWW/GCW comparisons used two-sided Mann–Whitney U tests with tie-adjusted normal approximations and continuity correction. GCW, GWW and GWW/GCW were analysed without covariate adjustment or multiplicity correction; their nominal p values are exploratory and do not provide confirmatory evidence for GWE. Adjusted effects are in percentage points, LBBAP minus RVP. A dash denotes an adjusted analysis not performed. CI, confidence interval; GCW, global constructive work; GWE, global myocardial work efficiency; GWW, global wasted work; LBBAP, left bundle branch area pacing; LVEF, left ventricular ejection fraction; RVP, right ventricular mid-septal pacing.

Table S6. Within-patient LVEF change from pre-implantation to extended follow-up.

**Group**	**Pre-implantation LVEF (%)**	**Extended follow-up LVEF (%)**	**Within-patient change, pp (95% CI)**	**Paired t-test p value**
LBBAP (n = 34)	57.19 ± 4.46	58.39 ± 4.73	+1.20 (−1.25 to +3.65)	0.327
RVP (n = 29)	58.50 ± 4.12	52.35 ± 6.64	−6.15 (−9.16 to −3.14)	<0.001

Values are mean ± standard deviation. CI, confidence interval; pp, percentage points. Within-group change was assessed by paired t-test; two-sided exact Wilcoxon signed-rank p values were 0.317 and <0.001, respectively. The between-group difference in change was +7.35 pp (95% CI 3.55 to 11.16; Welch p < 0.001; Mann–Whitney p < 0.001), and +7.00 pp (95% CI 3.12 to 10.88, p < 0.001) after adjustment for age, sex, hypertension and atrial fibrillation. Both within-group and between-group change analyses are exploratory and descriptive because follow-up duration differed among participants and no common-time longitudinal model was fitted. LBBAP, left bundle branch area pacing; LVEF, left ventricular ejection fraction; RVP, right ventricular mid-septal pacing.

Table S7. Sensitivity analyses for alternative covariates, calendar time and follow-up duration.

Model	GWE: β (95% CI), p	LVEF: β (95% CI), p
Primary: age, sex, hypertension, AF	+4.96 (0.87 to 9.04), 0.018	+5.73 (2.77 to 8.68), <0.001
Diabetes mellitus substituted for AF	+4.92 (0.72 to 9.11), 0.022	+6.05 (3.10 to 9.00), <0.001
Dyslipidaemia substituted for AF	+4.45 (0.19 to 8.70), 0.041	+5.69 (2.66 to 8.72), <0.001
Moderate tricuspid regurgitation substituted for AF	+4.78 (0.52 to 9.05), 0.029	+5.45 (2.45 to 8.46), <0.001
Pacing indication substituted for AF	+4.47 (0.22 to 8.72), 0.039	+5.73 (2.71 to 8.75), <0.001
Pre-implantation LVEF substituted for AF	+4.79 (0.59 to 8.99), 0.026	+5.61 (2.64 to 8.58), <0.001
Primary model plus pre-implantation LVEF	+5.05 (0.88 to 9.21), 0.018	+5.57 (2.57 to 8.57), <0.001
Follow-up duration substituted for AF	+4.76 (0.58 to 8.93), 0.026	+5.63 (2.69 to 8.57), <0.001
Primary model plus implantation year	+4.97 (0.87 to 9.07), 0.018	+5.74 (2.79 to 8.69), <0.001
Primary model plus implantation era	+4.91 (0.79 to 9.03), 0.020	+5.61 (2.71 to 8.52), <0.001
Restricted to 12–36 months (n = 43)	+3.96 (−0.56 to 8.48), 0.084	+4.31 (0.38 to 8.24), 0.032
Restricted to 18–36 months (n = 32)	+3.55 (−2.64 to 9.73), 0.249	+3.49 (−0.73 to 7.71), 0.101

Values are adjusted LBBAP-minus-RVP differences (β) in percentage points, with 95% CIs and p values. All models include pacing strategy and use complete cases; CIs and p values use the Student t distribution. Each substitution model retains pacing strategy, age, sex and hypertension and replaces atrial fibrillation with the named covariate. The pre-implantation LVEF substitution model replaces atrial fibrillation with pre-implantation LVEF, whereas the addition model (primary model plus pre-implantation LVEF) adds pre-implantation LVEF to all four primary covariates; both are shown in this table. Implantation year was entered as a continuous calendar-year covariate; implantation era was defined as 2021-2022 versus 2023-2024, with 2021-2022 as the reference. Full-cohort models use n = 63; window restrictions use the n shown in the row. Across the six substitution models, GWE estimates differ from the primary estimate by at most 0.51 percentage points. This sensitivity assessment does not exclude residual confounding or establish that any candidate covariate is unrelated to the outcome. All sensitivity p values are nominal and uncorrected for multiplicity. AF, atrial fibrillation; CI, confidence interval; GWE, global myocardial work efficiency; LBBAP, left bundle branch area pacing; LVEF, left ventricular ejection fraction; RVP, right ventricular mid-septal pacing.

Table S8. Standardised differences in participant characteristics, implantation year and follow-up duration.

Variable	LBBAP (n = 34)	RVP (n = 29)	Std. diff
Hypothyroidism	0 (0%)	4 (14%)	−0.566
Former smoker	0 (0%)	4 (14%)	−0.566
Dyslipidaemia	11 (32%)	16 (55%)	−0.473
Moderate tricuspid regurgitation	5 (15%)	10 (34%)	−0.472
Aspirin	14 (41%)	6 (21%)	+0.455
Second-degree AV block	19 (56%)	10 (34%)	+0.440
Third-degree AV block	15 (44%)	19 (66%)	−0.440
Body mass index, kg/m²	28.7 ± 3.8	27.1 ± 4.1	+0.405
Beta-blocker	14 (41%)	17 (59%)	−0.354
Diabetes mellitus	10 (29%)	13 (45%)	−0.323
Pre-implantation LVEF, %	57.19 ± 4.46	58.50 ± 4.12	−0.305
Peripheral artery disease	7 (21%)	3 (10%)	+0.286
Hypertension	15 (44%)	16 (55%)	−0.222
Male sex	20 (59%)	20 (69%)	−0.212
Follow-up duration, years	2.04 ± 0.94	2.22 ± 0.92	−0.194
COPD	2 (6%)	3 (10%)	−0.164
Anticoagulant	2 (6%)	3 (10%)	−0.164
ACEi/ARB	12 (35%)	12 (41%)	−0.125
Chronic kidney disease	2 (6%)	1 (3%)	+0.116
Current smoker	2 (6%)	1 (3%)	+0.116
Creatinine, mg/dL	1.3 ± 1.2	1.2 ± 0.6	+0.105
eGFR, mL/min/1.73 m²	56.2 ± 25.4	58.3 ± 22.8	−0.087
Age, years	76.12 ± 8.24	76.58 ± 10.47	−0.048
Intrinsic QRS duration, ms	112.4 ± 25.8	110.7 ± 24.3	+0.068
Atrial fibrillation	4 (12%)	4 (14%)	−0.061
Coronary artery disease	1 (3%)	1 (3%)	−0.029
Prior heart failure	1 (3%)	1 (3%)	−0.029
Prior stroke/TIA	1 (3%)	1 (3%)	−0.029
Implantation year	2022.44 ± 0.99	2022.41 ± 1.02	+0.027
P2Y12 inhibitor	7 (21%)	6 (21%)	−0.003

Standardised differences describe measured balance; positive values indicate higher values or prevalence in the LBBAP group. Continuous differences use the mean difference divided by the square root of the average of the two within-group sample variances; binary differences use the proportion difference divided by the square root of the mean of the two group-specific Bernoulli variances. Absolute values above 0.10 and 0.25 are descriptive indications of imbalance and notable imbalance, respectively. Of the 30 displayed rows, 21 exceed 0.10 and 12 exceed 0.25. The rows include both mutually exclusive categories of pacing indication, implantation year and follow-up duration, so they are not 30 independent baseline variables. Table S7 presents substitution analyses for diabetes, dyslipidaemia, moderate tricuspid regurgitation, pacing indication, baseline LVEF and follow-up duration; it does not separately adjust for every imbalanced characteristic. These analyses cannot exclude residual confounding by measured or unmeasured factors. ACEi, angiotensin-converting enzyme inhibitor; ARB, angiotensin receptor blocker; AV, atrioventricular; COPD, chronic obstructive pulmonary disease; eGFR, estimated glomerular filtration rate; LBBAP, left bundle branch area pacing; LVEF, left ventricular ejection fraction; RVP, right ventricular mid-septal pacing; TIA, transient ischaemic attack.

Table S9. Propensity-score sensitivity analyses of GWE and LVEF.

Approach	GWE: β (95% CI), p	LVEF: β (95% CI), p
Primary ANCOVA (reference)	+4.96 (0.87 to 9.04), 0.018	+5.73 (2.77 to 8.68), <0.001
Propensity score as covariate	+5.10 (0.40 to 9.81), 0.034	+5.57 (2.28 to 8.85), 0.001
Propensity-score quintile strata	+4.15 (−0.69 to 9.00), 0.092	+5.03 (1.65 to 8.41), 0.004
Stabilised IPTW, robust SE	+5.33 (0.80 to 9.86), 0.021	+6.53 (3.33 to 9.74), <0.001
Common support (n = 57; 28 LBBAP/29 RVP)	+3.69 (−0.54 to 7.93), 0.086	+4.78 (1.76 to 7.81), 0.003

The propensity score for initial LBBAP assignment was estimated by logistic regression with age, sex, hypertension, atrial fibrillation, diabetes, dyslipidaemia, moderate tricuspid regurgitation, pacing indication and pre-implantation LVEF. McFadden pseudo-R^2 was 0.170 and the likelihood-ratio p value was 0.097; neither establishes absence of selection bias. Common support was the interval between the larger arm-specific minimum and the smaller arm-specific maximum (0.111 to 0.885), retaining 57 of 63 patients: 28 LBBAP and 29 RVP. Six LBBAP patients and no RVP patients were excluded. Stabilised weights were $P(T=1)/e$ for LBBAP and $P(T=0)/(1-e)$ for RVP; raw weights ranged from 0.52 to 6.24. The weighted model used weights capped at the pooled 99th percentile (R type-7 quantile; 4.86, affecting one observation), HC3 standard errors, and normal-Wald 95% CIs and p values. Kish effective sample sizes were 21.9 for LBBAP and 19.9 for RVP; the pooled value was 41.7. The largest individual weight accounted for 13.7% of total LBBAP weight and 14.3% of total RVP weight. The mean absolute standardised difference fell from 0.284 to 0.074, but age (+0.11) and pre-implantation LVEF (+0.18) remained above 0.10; age imbalance increased in magnitude. Individual coefficients, balance, stratum composition and variance conventions are in Table S11. LVEF estimates were positive with all reported CIs above zero. GWE CIs included zero for quintile stratification and common-support restriction. The common-support GWE estimate was +3.69 versus +4.96 percentage points in the primary model. These exploratory analyses use nominal p values and cannot exclude residual confounding; limited overlap, weight concentration and sample size limit their precision. β, adjusted LBBAP-minus-RVP difference in percentage points; CI, confidence interval; GWE, global myocardial work efficiency; HC3, heteroscedasticity-consistent type 3 covariance estimator; IPTW, inverse-probability-of-treatment weighting; LBBAP, left bundle branch area pacing; LVEF, left ventricular ejection fraction; RVP, right ventricular mid-septal pacing; SE, standard error.

Table S10. Descriptive standardised mean differences for echocardiographic endpoints.

**Endpoint**	**LBBAP (n = 34)**	**RVP (n = 29)**	**Standardised mean difference**
LVEF, %	58.39 ± 4.73	52.35 ± 6.64	1.05
GWE, %	85.23 ± 6.82	80.00 ± 9.41	0.64
GCW, mmHg%	1809 ± 437	1582 ± 359	+0.57
GWW/GCW ratio	0.165 ± 0.090	0.236 ± 0.165	−0.54
GWW, mmHg%	281 ± 137	341 ± 182	−0.37
GLS, %	−16.9 ± 2.2	−16.4 ± 2.9	−0.19
LVEDD, mm	48.1 ± 5.53	48.9 ± 5.49	−0.15
PSD, ms	61.9 ± 15.23	63.5 ± 18.3	−0.10
LVESD, mm	34.8 ± 5.31	35.2 ± 5.51	−0.07

Values are observed mean ± standard deviation. For GWE, LVEF, GCW, GWW and GWW/GCW, standardised mean differences were calculated from unrounded patient-level values. The standardised mean difference is the LBBAP-minus-RVP mean difference divided by $\sqrt{[(SD^2 LBBAP + SD^2 RVP)/2]}$ and is not an adjusted treatment effect. These estimates do not establish the study's prospective power. GLS differed by −0.50 percentage points, with a reported 95% confidence interval from −1.82 to +0.82; this interval includes zero and differences in either direction. Nonsignificant comparisons do not establish equivalence. Inferential p values and adjusted estimates are reported in the relevant outcome tables. GCW, global constructive work; GLS, global longitudinal strain; GWE, global myocardial work efficiency; GWW, global wasted work; LVEDD, left ventricular end-diastolic diameter; LVEF, left ventricular ejection fraction; LVESD, left ventricular end-systolic diameter; PSD, peak strain dispersion.

Table S11. Propensity-score coefficients and covariate balance before and after weighting.

**Covariate**	**Score model coefficient (SE)**	**Std. diff before**	**Std. diff after**
Age, years	−0.028 (0.037)	−0.048	+0.107
Male sex	−0.467 (0.659)	−0.212	−0.090
Hypertension	−0.117 (0.662)	−0.222	−0.068
Atrial fibrillation	−0.937 (0.969)	−0.061	−0.022
Diabetes mellitus	−0.988 (0.662)	−0.323	+0.044
Dyslipidaemia	−1.120 (0.602)	−0.473	−0.061
Moderate tricuspid regurgitation	−1.068 (0.718)	−0.472	+0.062
Third-degree AV block	−1.098 (0.635)	−0.440	+0.037
Pre-implantation LVEF, %	−0.084 (0.071)	−0.305	+0.179
Mean absolute value	-	0.284	0.074
Maximum absolute value	-	0.473	0.179

Logistic model for initial LBBAP assignment (n = 63) with nine covariates; converged with finite coefficient estimates. Intercept +9.391 (SE 5.045). Log-likelihood −36.07 versus null −43.47; McFadden pseudo-R^2 0.170; likelihood-ratio $\chi^2(9) = 14.79$, p = 0.097. Standardised differences were the LBBAP-minus-RVP mean or proportion difference divided by the square root of the average within-group variance. For continuous covariates, unweighted differences used sample variances and weighted differences used population variances calculated as $\Sigma w(x - \text{weighted mean})^2/\Sigma w$; binary variances were $p(1 - p)$, using weighted proportions after weighting. Positive values indicate higher values or prevalence in LBBAP. Weighted values use stabilised weights capped at the pooled type-7 99th percentile (4.86; one observation). Age and baseline LVEF remained above an absolute difference of 0.10. Quintiles 1–5 contained 10, 8, 4, 6 and 1 RVP patients, and 3, 4, 9, 6 and 12 LBBAP patients; the highest quintile had one RVP patient. Kish effective sample size, $(\Sigma w)^2/\Sigma w^2$, was 21.9 for LBBAP and 19.9 for RVP; the pooled value was 41.7. AV, atrioventricular; LBBAP, left bundle branch area pacing; LVEF, left ventricular ejection fraction; RVP, right ventricular mid-septal pacing; SE, standard error.

Section S3. Supplementary Figures

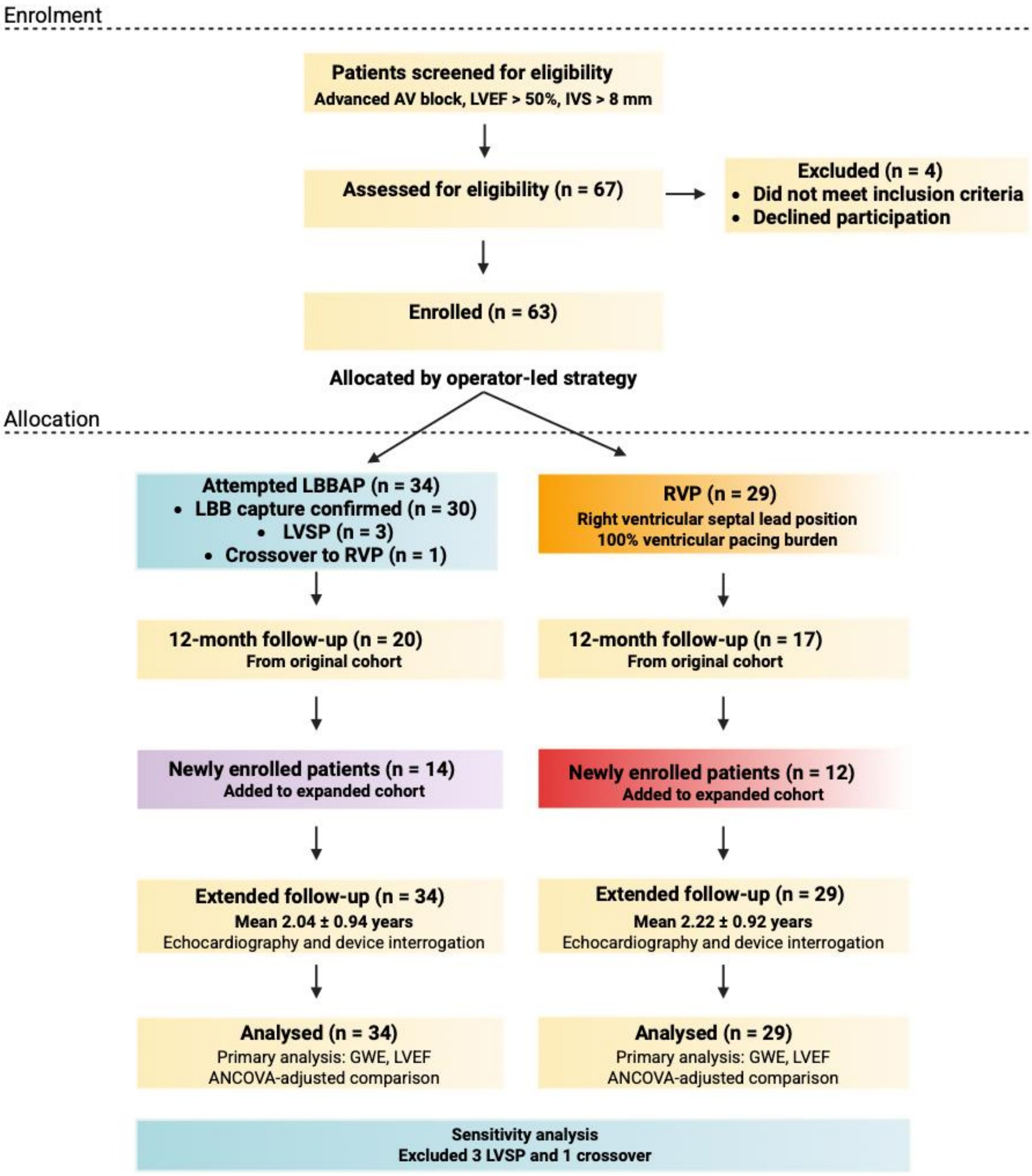

Figure S1. Patient flow diagram. Overall, 63 patients were enrolled, including 34 allocated to left bundle branch area pacing (LBBAP) and 29 to right ventricular pacing (RVP). Within the LBBAP group, 30 patients fulfilled left bundle branch pacing (LBBP) criteria, 3 were classified as left ventricular septal pacing (LVSP), and 1 crossed over to RVP. The original study enrolled 38 patients; its 12-month analysis included 37 completers and has been published previously. The extended follow-up included the full expanded cohort (n = 63), with echocardiographic data available for all patients. GWE is the primary endpoint and LVEF the principal adjusted secondary endpoint. Figure created in BioRender. Bozika, M. (2026).

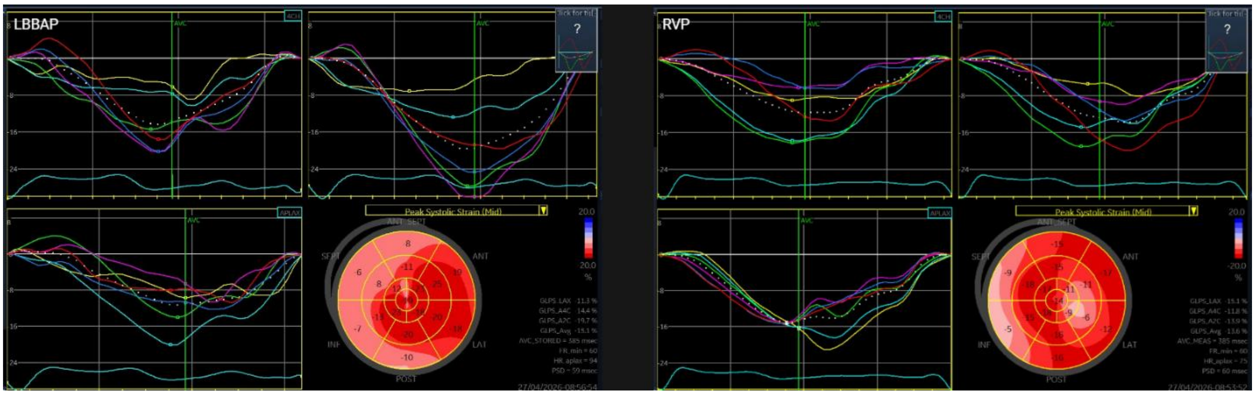

Figure S2. Representative two-dimensional speckle-tracking longitudinal strain analysis in patients undergoing left bundle branch area pacing and right ventricular pacing. Colour-coded strain curves correspond to left ventricular segments derived from the apical four-chamber, apical two-chamber, and apical long-axis views. Dots on each curve indicate peak systolic strain, and the green vertical line denotes aortic valve closure at end-systole. The bull's-eye plot displays global and segmental peak systolic longitudinal strain. In the representative LBBAP patient, average GLS was -15.1% and PSD was 59 ms. In the representative RVP patient, average GLS was -13.6% and PSD was 60 ms. These examples illustrate the derivation of GLS and PSD from segmental strain curves and are not intended to represent group-level differences.

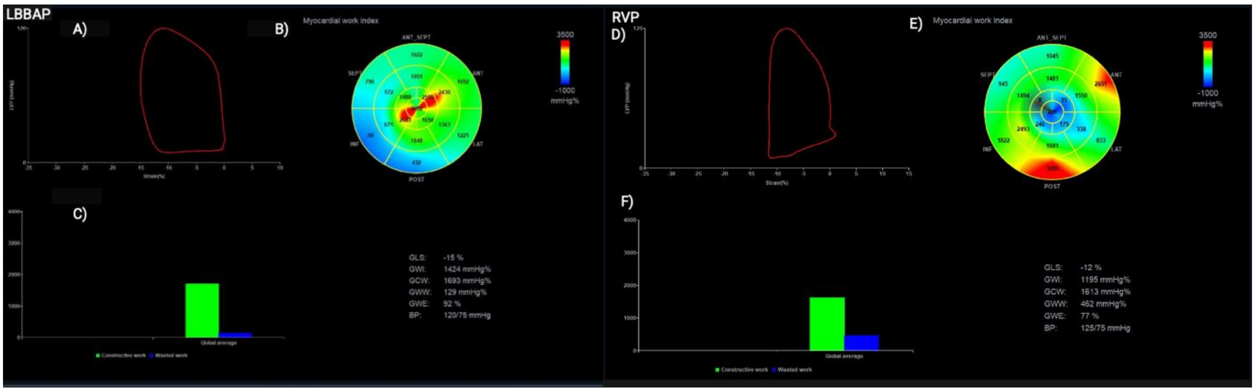

Figure S3. Representative myocardial work analysis in patients undergoing left bundle branch area pacing and right ventricular pacing. Panels A-C show myocardial work analysis in a representative LBBAP patient. (A) Global left ventricular pressure-strain loop. (B) Bull's-eye display of segmental myocardial work index. (C) Distribution of constructive myocardial work and wasted myocardial work. In this representative LBBAP patient, GLS was -15%, GWI was 1424 mmHg%, GCW was 1693 mmHg%, GWW was 129 mmHg%, and GWE was 92%, with brachial blood pressure of 120/75 mmHg. Panels D-F show myocardial work analysis in a representative RVP patient. (D) Global left ventricular pressure-strain loop. (E) Bull's-eye display of segmental myocardial work index. (F) Distribution of constructive myocardial work and wasted myocardial work. In this representative RVP patient, GLS was -12%, GWI was 1195 mmHg%, GCW was 1613 mmHg%, GWW was 462 mmHg%, and GWE was 77%, with brachial blood pressure of 125/75 mmHg. These representative examples illustrate the derivation of myocardial work indices and should not be interpreted as individual-level evidence of the between-group differences reported in the cohort analysis.
